# Viral genomic methylation and the interspecies evolutionary relationships of ranavirus

**DOI:** 10.1371/journal.ppat.1012736

**Published:** 2024-11-25

**Authors:** Weiqiang Pan, Mincong Liang, Yanlin You, Zhimin Li, Shaoping Weng, Jianguo He, Changjun Guo

**Affiliations:** School of Marine Sciences, State Key Laboratory for Biocontrol / Southern Marine Science and Engineering Guangdong Laboratory (Zhuhai), Guangdong Province Key Laboratory of Aquatic Economic Animals & Guangdong Provincial Observation and Research Station for Marine Ranching of the Lingdingyang Bay, Sun Yat-sen University, Guangzhou, Guangdong, China; Virginia Tech: Virginia Polytechnic Institute and State University, UNITED STATES OF AMERICA

## Abstract

Ranaviruses are capable of infecting both wild and farmed fish, amphibians, and reptiles, leading to significant economic losses and ecological risks. Currently, ranaviruses have been found in at least 175 species spanning six continents. Except for Singapore grouper iridovirus (SGIV), ranavirus genomes are generally regarded as highly methylated. Nevertheless, our comprehension of the methylation characteristics within ranaviruses remains limited. Despite the numerous genomes currently included in the GenBank database, a complete phylogenetic tree for ranaviruses has not yet been determined, and interspecific evolutionary relationships among ranaviruses have not been thoroughly investigated. In this study, the whole-genome methylation profile of mandarin fish ranavirus (MRV; a ranavirus) was investigated, revealing a methylation level of 16.04%, and hypomethylation of the MRV genome was detrimental to viral replication, speculating the genome methylation may play an important role in MRV replication. Furthermore, by combining with whole-genome DNA sequence phylogenetic analyses, we propose the possibility of an interspecies evolutionary relationship among ranaviruses, with the presence of four distinct evolutionary lineages within ranavirus evolution: "SGIV, SCRAV(MRV/LMBV), EHNV/ENARV/ATV, and CMTV/FV3", which might be also supported by the genomic collinearity, natural host range and host habitats. Furthermore, ranavirus genomic methylation levels may provide additional evidence for this hypothesis, but further proof is needed. Our work enhances the understanding of the role of genome methylation in ranaviruses and is beneficial for the prevention and control of ranavirus diseases; simultaneously, the proposed evolutionary hypothesis of ranavirus provides novel insights and ideas for exploring the evolutionary trajectory of viruses.

## Author summary

Incidents of ranavirus infections have been reported in at least 175 species of metamorphic vertebrates across six continents, including fish, amphibians, and reptiles, posing a significant threat to both wild and farmed animals and causing substantial losses to economic animal farming. Currently, the diversity and complexity of ranavirus taxonomy remain incompletely understood, and the interspecific evolutionary relationships of ranavirus have not been fully elucidated. In this study, we demonstrate that the MRV genome exhibits high levels of methylation, approximately 16.04%, which plays a pivotal role in the viral replication process. The whole-genome phylogenetic tree, genomic collinearity, natural host range, and host habitats collectively support the "SGIV, SCRAV(MRV/LMBV), EHNV/ENARV/ATV, and CMTV/FV3" hypothesis of ranavirus interspecies evolutionary relationships. Furthermore, we speculate genome methylation level may serve as evidence for the hypothesis. These findings are anticipated to provide novel insights into the taxonomic and evolutionary relationships of ranavirus and facilitate the control of the ranavirus diseases.

## Introduction

Ranavirosis, an emerging infectious disease with globally implications, is caused by a group of viruses belonging to the *Ranavirus* genus within the *Iridoviridae* family [1,2]. Characterized by a brief incubation period and exceptionally high mortality rates [3], this disease frequently leads to mass mortality among both wild and farmed fish, amphibians, and reptiles [2,4–6]. With reported cases spanning across North and South America, Europe, Asia, Africa, and Australia, over 175 animal species have been infected with these viruses [2,4,6,7]. The World Organisation for Animal Health has designated ranavirosis as a notifiable disease, emphasizing its significance and the need for rigorous monitoring [6]. Given the considerable threat posed by ranaviruses to these animals and their associated industries, urgent research is necessitated to enhance our comprehension of the disease and develop effective prevention strategies.

Ranaviruses are large, double-stranded DNA viruses characterized by a circularly permuted, terminally redundant genome with a size ranging from 104 to 140 kbp and possessing G + C contents of 49% to 57% [8,9]. With the exception of the Singapore grouper iridovirus (SGIV), all ranavirus genomes are considered to have high levels of methylation due to a virus-encoded DNA cytosine methyltransferase (DNMT) [1,10]. DNA methylation serves as a regulatory mechanism for replication in numerous DNA viruses, such as the Epstein–Barr virus, where methylation status modulates gene expression [10]. Recent studies have indicated that genome methylation of the infectious spleen and kidney necrosis virus (ISKNV, a megalocytivirus) is crucial for evading host immune responses [11]. Earlier research showed that over 20% of cytosine residues in frog virus 3 (FV3) DNA were methylated [12]. Despite decades of research [13], the methylation sites, distribution, and characteristics of ranaviruses remain unknown.

According to the report from the International Committee on Taxonomy of Viruses (ICTV), the *Ranavirus* genus encompasses viruses from seven species [1]. However, despite the substantial surge in reports of ranavirus infections, the taxonomic classification of these viruses remains contentious. Furthermore, the escalation in international commercial trade involving amphibians, reptiles, and fish, the expansion of aquaculture, and the increased introduction of aquaculture-economical species across regions have facilitated a more rapid and extensive dissemination of ranaviruses, resulting in an elevated incidence of novel infections [14]. Extensive research is advancing to better define and the classification of ranaviruses to better define and differentiate virus taxa within the *Ranavirus* genus. While the evolutionary history of ranavirus is currently under investigation, it appears to have undergone at least several host group "jumps" (e.g. fish to frog) [15]. The controversy surrounding species division may also arise from this phenomenon. Recent studies have indicated that a set of nine core genes can serve as markers for identifying unknown iridoviruses at the genus level [16]. However, the sequence of species differentiation within the *Ranavirus* genus remains to be elucidated.

Mandarin fish ranavirus (MRV), a member of the *Ranavirus* genus in the *Iridoviridae* family, was first reported in 2017 from adult hybrid mandarin fish (*Siniperca chuatsi*) [17]. Shortly afterward, widespread outbreaks occurred in Guangdong, China’s primary mandarin fish farming region, resulting in alarming mortality rates of up to 96.2% [18]. Symptoms associated with MRV infection include abdominal swelling, ascites, and exophthalmos [17]. However, comprehension of its pathogenic mechanisms and prevention strategies is still limited, necessitating further research.

In this study, a comprehensive analysis of the whole-genome methylation landscape of MRV was conducted, examining the sites, distribution, and features of methylation within the MRV genome. The findings are expected to enhance our understanding of the role of genome methylation in ranavirus infections. In addition, a comprehensive phylogenetic tree analysis based on whole-genome was conducted across all ranavirus species, enhancing taxonomic and evolutionary studies.

## Results

### Genome organization of MRV virion

**Fig 1:**
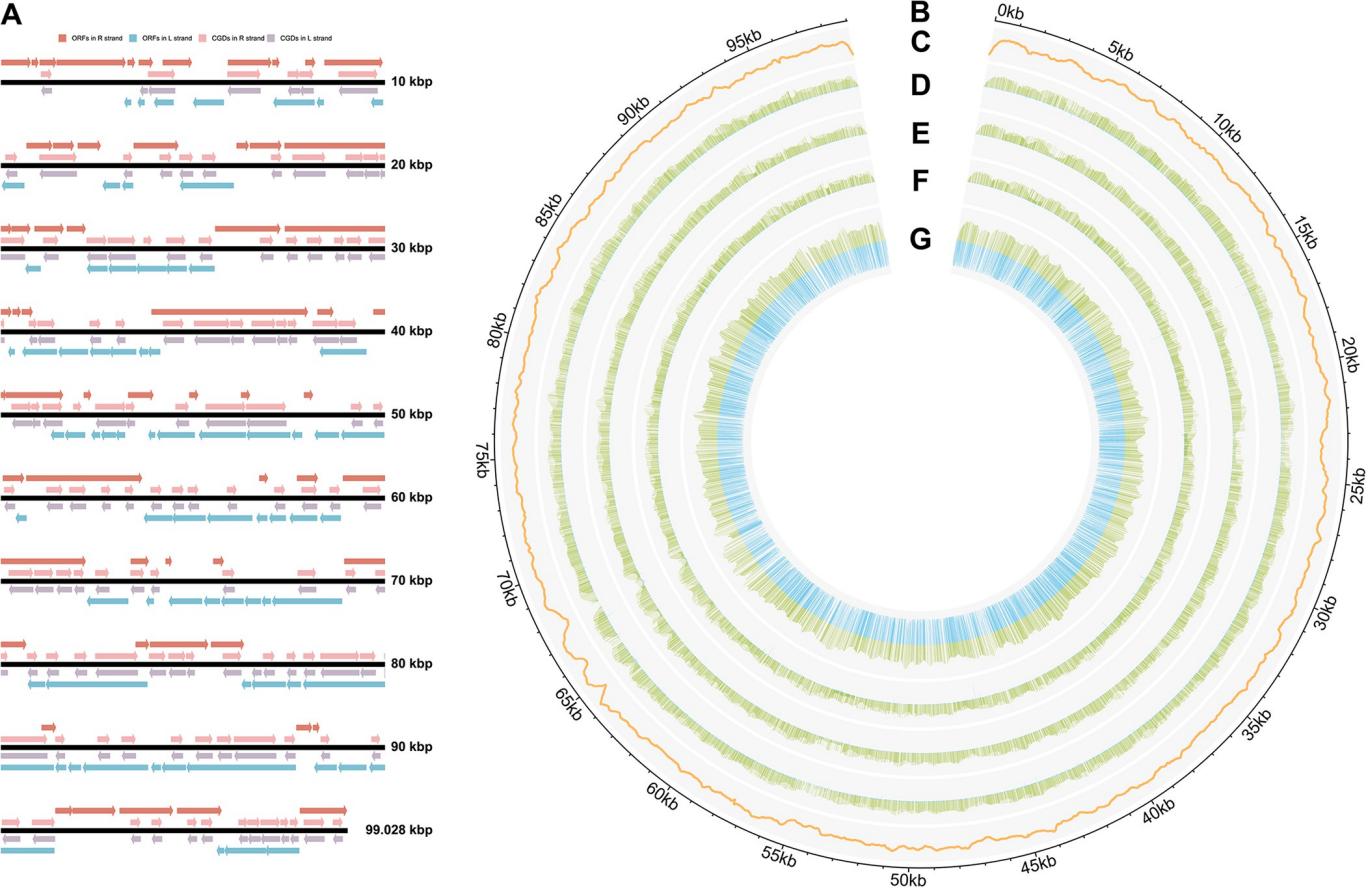
Organization and hypermethylated landscape of the MRV genome. (A) The distribution of ORFs and CGDs in the MRV genome. Arrows indicate the location, orientation, and putative size represent each ORF or CGD. Light corals and skyblue arrows indicate ORFs located on the R and L strands, respectively, and light pink and thistle arrows indicate CGDs located on the R and L strands, respectively. (B) Depiction of the MRV genome. (C) Sum of sequencing depth of WGBS at each position (*n* = 4). (D–G) The yellow-green hue indicates the aggregate counts of methylated and unmethylated bases in whole-genome bisulfite sequencing (WGBS), while the blue color denotes the average methylation level at each CA (D), CT (E), CC (F), or CG (G) site (*n* = 4). Detailed numerical data are available in S5 and S6 Data.

The MRV virion was purified, and the viral DNA was subsequently sequenced using the Illumina NovaSeq 6000 (PE150). Following rigorous data processing and analysis, the MRV genome, spanning 99,028 bp (Accession number: MG941005.3), was obtained. This genome exhibits a G + C content of 52.11%. Approximately 125 presumptive ORFs encoding polypeptides ranging from 50 to 1354 amino acid residues were identified (S1 Data). Among these, 54 ORFs reside on the R strand, while 71 ORFs reside on the L strand. Fig 1A depicts the relative positions of these putative ORFs within the genome. The 125 predicted ORFs constitute 95% of the genetic information encoded by the MRV genome, distributed across both strands (44% forward, 56% reverse) (Fig 1A). The ORFs of MRV are arranged in a relatively compact manner, with 68 overlapping and 57 non-overlapping ORFs. The average distance between non-overlapping ORFs is approximately 90 bp, ranging from 0 bp to 314 bp. Furthermore, 39 ORFs were identified as possessing conserved domains or motif.

The MRV genome in the GenBank database has been updated through whole-genome sequencing. Compared to the original genome (Accession number: MG941005.1), this study reveals an additional 3,054 bp. Furthermore, 23 novel ORFs have been annotated, with predicted potential protein structures or functions encompassing cytosine DNA methyltransferase, tyrosine kinase, D5 family NTPase/ATPase, interleukin-1 beta convertase precursor, DNA repair protein RAD2, thiol oxidoreductase, major capsid protein, and others (S1 Data). The findings from the aforementioned resequencing analysis of the MRV genome have significantly deepened our understanding of its genetic coding capacity.

### Methylation patterns of the MRV genome

**Fig 2:**
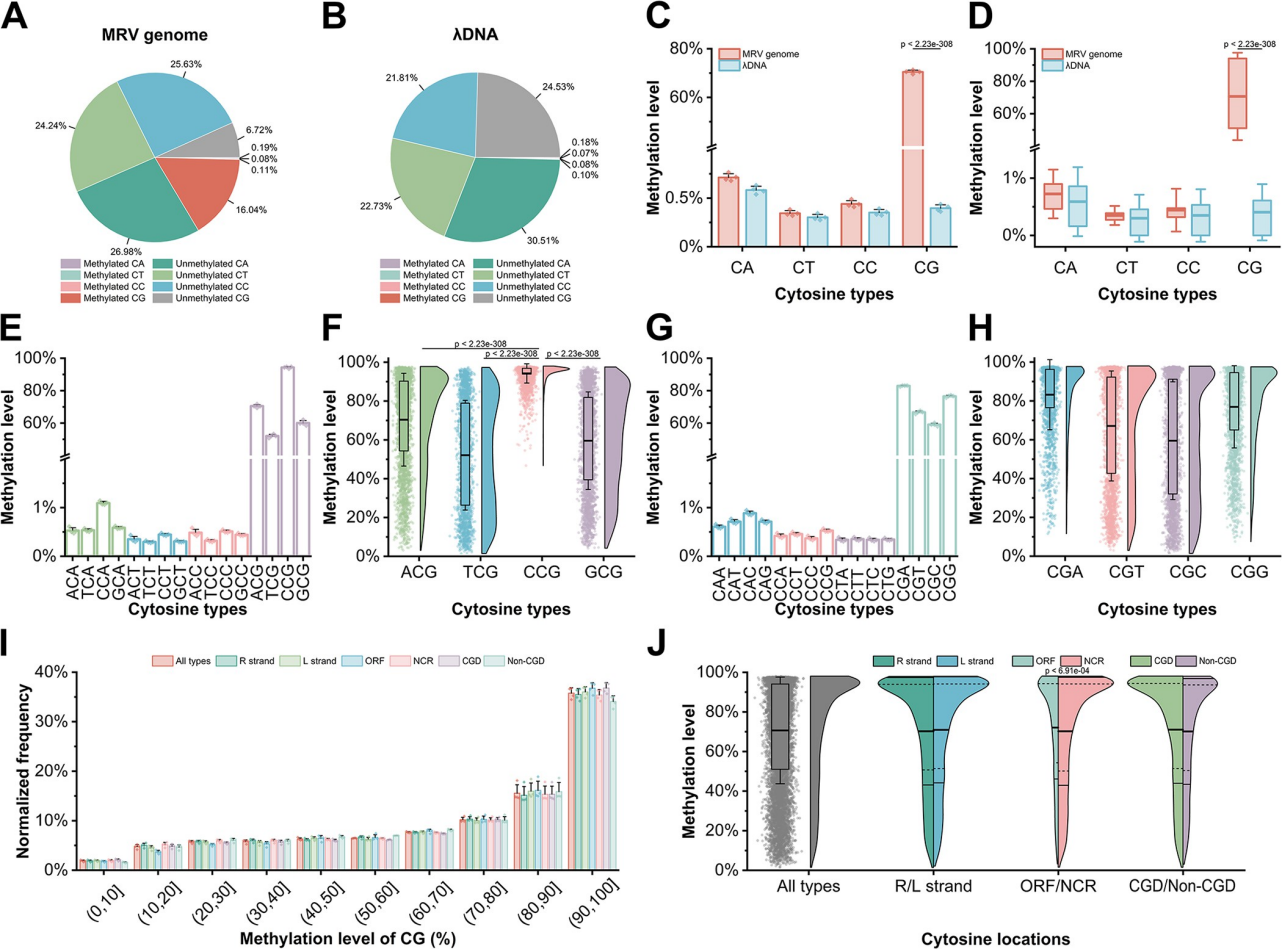
The high methylation levels of the MRV genome are illustrated, highlighting CpG sites exhibiting elevated methylation across all contexts. (A) The average percentages of methylated and unmethylated cytosine in the MRV derived from WGBS data analyzed by Bismark are presented (*n* = 4). (B) The average percentages of methylated and unmethylated cytosine in the λDNA derived from WGBS data analyzed by Bismark are presented (*n* = 4). (C) The methylation level in CA, CT, CC, and CG contexts were quantified (*n* = 4). (D) The distribution of methylation levels at each site of CA, CT, CC, and CG. (*n* = 4). (E) Cytosine methylation levels grouped by the 5’ region nucleotides were shown (*n* = 4). (F) A focus is placed on CpG context methylation levels, grouped by the 5’ nucleotides (*n* = 4). (G) Cytosine methylation levels grouped by the subsequent one nucleotide in the 3’ region is presented (*n* = 4). (H) CpG contexts methylation levels, grouped by the same 3’ region nucleotides were specifically highlighted (*n* = 4). (I) The detailed distribution of methylation levels in the CG contexts (*n* = 4). (J) A comparative analysis of the distribution of highly methylated cytosines (*n* = 4). CGD stands for CG-dense regions. Solid lines represent the mean, while dashed lines depict the quartiles in (F, H, J). In (F, H, J), the box is delimited by the upper to the lower quartiles. Detailed numerical data are available in S6 Data.

To map site-by-site methylation landscape of MRV genome, bisulfite conversion and whole-genome sequencing were executed (Fig 1B–1G). From four duplicate samples, an average 36,482.38x cytosine was obtained for the site-specific methylation landscape of the MRV genome (Fig 1C). Methylation predominantly occurred in CG contexts within MRV genome’s CA, CT, CC, and CG sequences (Fig 1D–1G). Whole-genome bisulfite conversion sequencing showed that the effects of sequencing background noise has not yet been ruled out, with approximately 16.44% of cytosines in the MRV genome were methylated as 5-methylcytosine (5mC), while 83.57% remained unmethylated (Fig 2A). The unmethylated λDNA used as a negative control exhibited a background methylation level of merely 0.42%, indicating a conversion rate exceeding 99.5% (Fig 2B). Cytosines were categorized into CA, CT, CC, and CG contexts, with frequencies of approximately 27.17%, 23.32%, 25.74%, and 22.76%, respectively (Fig 2A). Methylation primarily occurred in CG contexts, with a level of 70.48% (Fig 2C). No significant differences in methylation levels were observed among CA, CT, and CC contexts (respectively 0.71%, 0.34%, and 0.44%) compared to the negative control (λDNA), which may be attributed to background noise in the sequencing analysis (Fig 2C). This result indicates that cytosine residues in CA, CT, and CC contexts of the MRV genome either do not undergo methylation or exhibit extremely low levels of methylation, whereas methylation predominantly occurs at CG contexts, and the accurate methylation level of the MRV genome is represented solely by the methylation level in the CG context, which is 16.04% (Fig 2A). Furthermore, in these methylated CG contexts, the average methylation level at each site exceeds about 70% (Fig 2D).

Typically, the nucleotide types at the 1 bp flanking the CG context in the genome do not exhibit a significant association with cytosine methylation levels [11]; however, this relationship remains unclear in ranaviruses. Therefore, we conducted an in-depth analysis of cytosine methylation in HCH contexts, where H represents any base (A, T, C, or G). Within the HCH contexts, only HCG sequence exhibited high levels of methylation (Fig 2E). Interestingly, cytosine methylation in ACG, GCG, and TCG contexts ranged from 0% to 100%, whereas methylation levels in CCG contexts clustered between approximately 75% to 100% (Fig 2F). These observations indicate that nearly all cytosines within CG dinucleotide contexts are methylated when cytosine is positioned as the 1 bp nucleotide following the 5’ region of a CG dinucleotide. However, the methylation status of cytosine within the CG dinucleotide context is not influenced by the adjacent at the 3’ region (Fig 2G and 2H). In summary, the MRV genome exhibits 16.04% cytosine methylation, predominantly occurring within CG contexts. Furthermore, the nucleotide type located on the 5’ side of the CG contexts appears to have an unknown correlation with the level of CG methylation.

### Distribution of cytosine contexts in the MRV genome

To further elaborate on the genomic methylation characteristics of MRV, a thorough analysis was conducted to investigate the distribution of cytosine within its genome. Upon comprehensive examination, it was found that 226 CpG-dense regions (CGD) were present across the MRV genome, with an equal distribution of 113 on the R strand and 113 on the L strand (Fig 1A and S2 Data). The analysis revealed a spectrum of methylated levels among the methylation CpG sites within the MRV genome, with a significant fraction exhibiting methylation exceeding 70% (Fig 2I). The methylation levels of the R and L chains in the MRV genome are approximately equivalent, hovering around 50% for both. However, the methylation level in the non-coding regions was significantly higher than that in the ORF regions. No significant difference was observed between the CGD and non-CGD regions (Fig 2J). These results suggest that the distribution of cytosine does not exhibit significant differences between the R and L strands of the genome, nor between CGD and non-CGD; however, a notable difference in distribution is observed between open reading frames (ORFs) and non-coding regions, with a predominant concentration in the latter.

### Molecular identification of methylation on MRV genomic DNA

**Fig 3:**
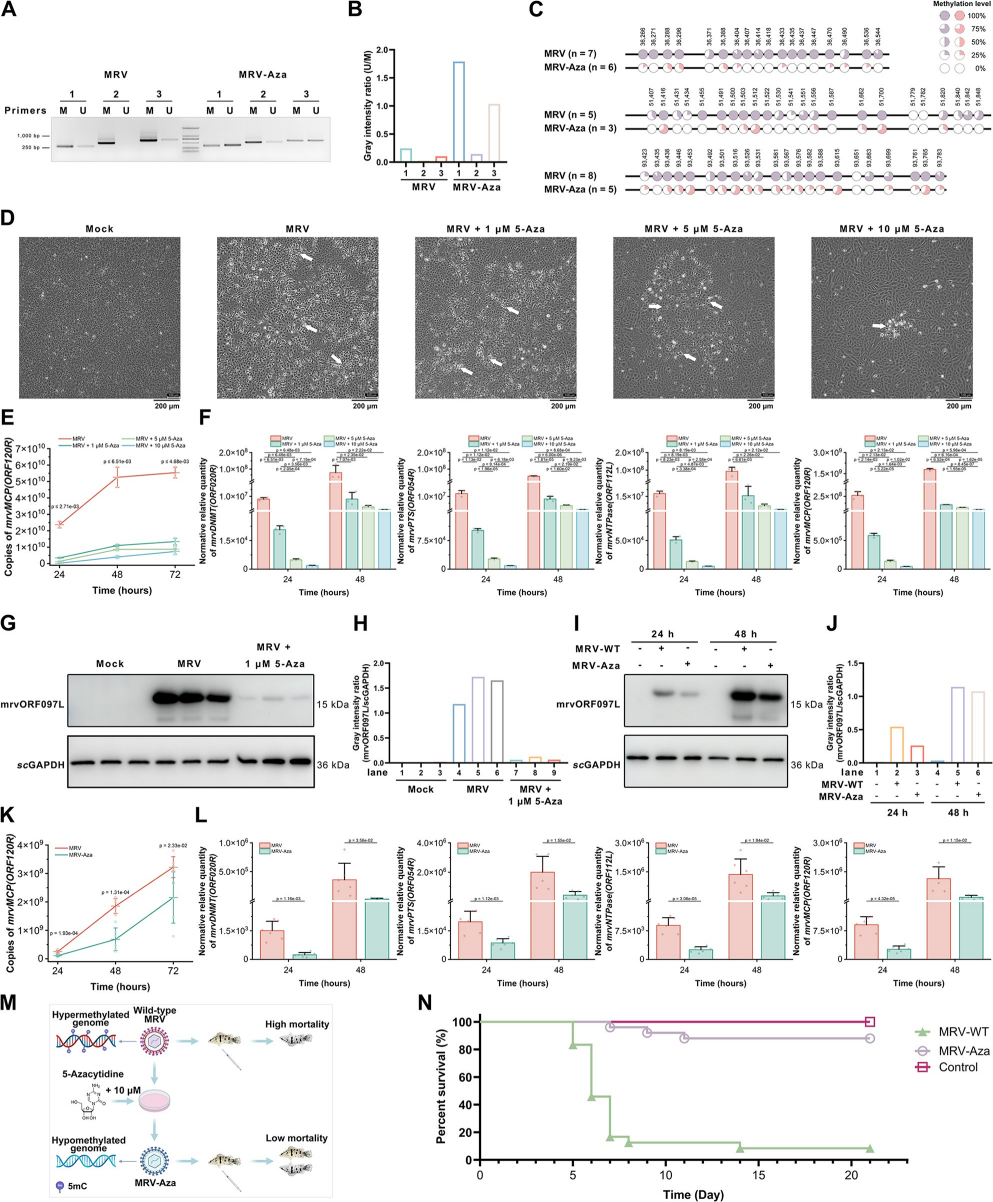
Molecular experiments have demonstrated that the MRV genome is highly methylated and critical for its replication. (A) The MSP results for three regions in the MRV and MRV-Aza genomes are presented. "M" indicates specific primers for methylated fragments, and "U" indicates specific primers for unmethylated fragments. (B) Gray density value analysis based on MSP results. (C) The BSP results for three regions in the MRV and MRV-Aza genomes reveal the methylation level of each CpG site in these regions. (D) The CPE induced by MRV in MFF-1 cells at 72 h post-infection was inhibited by 5-Azacytidine; The white arrow points to the CPE, and the black scale bars in the figure represent 200 μm. (E–F) Following the infection of MFF-1 cells with MRV (MOI  =  0.01) and different concentrations of 5-Azacytidine. (E) Total copies of MRV were quantified by qPCR at 24, 48, and 72 h post-infection. (F) Relative mRNA levels of mrv*ORF020R*, mrv*ORF054R*, mrv*ORF112L*, and mrv*ORF120R* were measured by RT-qPCR at 24 and 48 h post-infection. (G) Protein level of mrvORF097L was evaluated by western blotting at 48 h post-infection, and (H) gray density value analysis was also conducted. (I–L) Following the infection of MFF-1 cells with MRV and MRV-Aza at an MOI of 0.01. (I) Protein expression of mrvORF097L was evaluated by western blotting at 24 and 48 h post-infection, and (J) gray density value analysis was also performed. (K) Total copies of MRV were determined by qPCR at 24, 48 and 72 h post-infection. (L) Relative mRNA levels of mrv*ORF020R*, mrv*ORF054R*, mrv*ORF112L*, and mrv*ORF120R* were measured by RT-qPCR at 24 and 48 h post-infection. (M) A schematic representation of the reduction in MRV lethality resulting from demethylation. Created in BioRender. PAN, W. (2024) BioRender.com/h75e735. (N) Survival curves of *S*. *chuatsi* following intraperitoneal injection of MRV or MRV-Aza (*n* = 25). Data are presented as the mean ± SD (*n*  =  3). Detailed numerical data are available in S6 Data.

To validate whole-genome bisulfite sequencing (WGBS) data, BSP and MSP assays were conducted on the genomic DNA of the MRV virion. These techniques were employed to scrutinize three randomly selected sequences within the MRV genome. Both methodologies revealed the presence of methylated CpG sites within the MRV genome (MSP, Fig 3A; BSP, Fig 3C). Furthermore, MSP and BSP assays were conducted on virions (designated as MRV-Aza) derived from MFF-1 cells infected with MRV and treated with 5-Azacytidine. This compound, a widely used DNA methylation inhibitor, suppresses DNMT activity, resulting in decrease intracellular DNA methylation levels [19]. MSP analysis showed that specific primers targeting methylated fragments successfully amplified bands in MRV, whereas primers designed for non-methylated fragments yield a limited band. After treatment with 5-Azacytidine, specific primers for non-methylated fragments amplified a greater number of bands (Fig 3A). As shown in Fig 3B, MSP gray density analysis (U/M) of the MRV-Aza genome revealed a 7.3-, 6.4-, and 9.9-fold, increase in non-methylated fragment content for three random fragments, respectively, compared to wild-type MRV. In the BSP analysis, the methylation levels of CpG sites in the MRV-Aza genome were significantly lower than those in the untreated MRV genome. Specifically, the methylation levels of the three selected fragments were decreased to 0.12-, 0.24-, and 0.39-fold of the wild-type MRV, respectively, confirming the presence of 5mC methylation within the MRV genome (Fig 3C). These results indicate that the MRV genome is extensively methylated and that 5-Azacytidine treatment can effectively reduce its methylation levels.

### Hypomethylation of the MRV genome was disadvantageous for viral replication

DNA methylation has been reported as a regulatory mechanism employed by DNA viruses to modulate their replication cycles [10, 20]. Notably, genes encoding DNMT are present in the genomes of several viruses in the *Alphairidovirinae* subfamily, including MRV. Therefore, we further investigated the effect of 5-Azacytidine-induced genome hypomethylation on MRV replication. A significant inhibition of the apparent cytopathic effect (CPE) elicited by MRV in MFF-1 cells was observed following treatment with 5-Azacytidine (Fig 3D). This inhibitory effect increased with higher concentration of 5-Azacytidine. Additionally, the addition of 5-Azacytidine to MRV-infected MFF-1 cells resulted in significant suppression of MRV viral DNA (Fig 3E), RNAs (Fig 3F), and protein (Fig 3G and 3H). For example, after 24 h of treatment with 5-Azacytidine, viral copy numbers in the groups treated with 1, 5, and 10 μM concentrations were 0.15-, 0.06-, and 0.01-fold those of the untreated group, respectively (Fig 3E). Similar results were observed at the RNA level, where DNMT transcription levels were reduced to 0.0026-, 0.0006-, and 0.0002-fold those of the untreated group after treatment with 1, 5, and 10 μM of 5-Azacytidine, respectively (Fig 3F). At the protein level (Fig 3G), treatment with 1 μM of 5-Azacytidine for 24 h led to a 17.9-fold reduction in the level of MRV-encoded ORF097L protein (Fig 3H). These results suggested that the 5-Azacytidine treatment can suppress MRV replication. Furthermore, the duplication and transcription of the hypomethylated virions (MRV-Aza) were significantly inhibited compared to those of MRV (proteins, Fig 3I, 3J; DNA, Fig 3K; RNAs, Fig 3L). After infection with the same viral dose, the viral protein levels of ORF097L in cells infected with MRV-Aza decreased to 0.48- and 0.94-fold those of the WT-MRV group at 24 and 48 h post-infection, respectively (Fig 3I–1J). At the DNA level, the viral copy numbers of MRV-Aza were reduced to 0.43-, 0.37-, and 0.67-fold those of wild-type MRV at 24, 48, and 72 h post-infection, respectively (Fig 3K). Similarly, the gene transcription level of MRV-Aza was significantly lower than that of wild-type MRV, with DNMT level in the MRV-Aza-infected group being only 0.16- and 0.06-fold those in the wild-type MRV at 24 and 48 h post-infection, respectively (Fig 3L). The significance of genomic methylation for MRV was further demonstrated by a survival assay (Fig 3M). The survival rate of *S*. *chuatsi* injected with hypomethylated MRV (MRV-Aza) obtained via 5-Azacytidine treatment was significantly higher than that of wild-type MRV (Fig 3N). Specifically, the survival rate was 88% in the MRV-Aza group and 8.3% in the wild-type MRV group. These results suggested that the hypomethylation of the MRV genome was disadvantageous for viral replication, speculating the genome methylation may play an important role in MRV replication.

### Phylogenetic relationships between MRV and other iridoviruses

**Fig 4:**
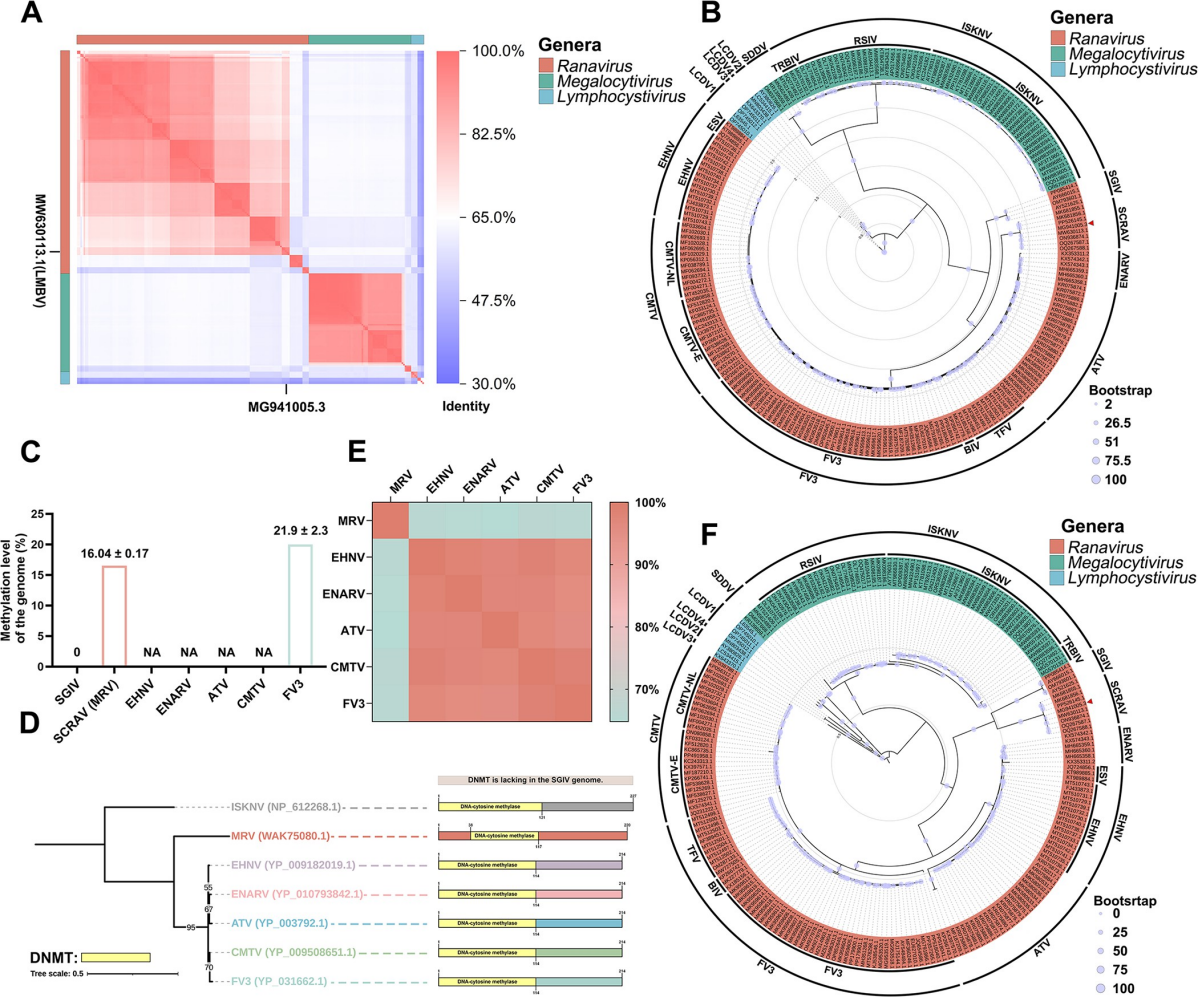
Phylogenetic analysis of MRV and *Ranavirus*. (A) Genomic alignment analysis was performed between MRV and other *Alphairidoviruses*. Detailed numerical data are available in S6 Data. (B) A phylogenetic tree of the virus was generated through an analysis of the amino acid sequences of multiple core proteins. This tree is distinguished by branches of different colors, representing different clusters. The red triangle indicates the position of MRV (MG941005.3). (C) A comparative analysis of the methylation levels of MRV genome with other published ranaviruses. (D) Structural domain prediction analysis of DNA methyltransferases (DNMTs) in prototypical species of ranaviruses. (E) The comprehensive analysis of the amino acid sequence similarity of the DNMT from Ranavirus was performed using the BLASTP tool. (F) A phylogenetic tree of iridoviruses was constructed based on whole genome sequences, with branches and outermost circles differentiated by various colors to represent different clusters. The red triangle indicates the position of MRV (MG941005.3).

To clarify the taxonomic status of MRV, a genomic nucleic acid similarity analysis was performed. The genomic nucleic acid sequences of a total of 217 strains of *Alphairidovirinae* from the GenBank database were used for this analysis (viruses were listed in S3 Data). Sequence identity analysis showed that the MRV genome sequence was more similar to that of the genus *Ranavirus* (Fig 4A), particularly to largemouth bass virus (LMBV), with a similarity of 95%. Subsequently, a protein evolutionary tree of the core genes of *Alphairidovirinae* was constructed (Fig 4B). The results showed that *Alphairidovirinae* was divided into three genera, concurring with the findings reported by ICTV. It is evident that MRV belongs to the genus *Ranavirus* in the phylogenetic tree. In the protein evolutionary tree of the core genes, ranaviruses could be distinctly classified into seven species, including Singapore grouper iridovirus (SGIV), Santee-Cooper ranavirus (SCRAV), European North Atlantic ranavirus (ENARV), epizootic haematopoietic necrosis virus (EHNV), ambystoma tigrinum virus (ATV), frog virus 3 (FV3), and common midwife toad virus (CMTV). Among them, EHNV encompassed two genotypes: European sheatfish virus (ESV) and EHNV; FV3 includes three genotypes: FV3, Bohle iridovirus (BIV), and tiger frog virus (TFV); and CMTV encompassed two genotypes: common midwife toad virus-E (CMTV-E) and common midwife toad virus-NL (CMTV-NL). Furthermore, the *Ranavirus* genus was classified into three sister lineages: SGIV (comprising SGIV), SCRAV (encompassing SCRAV, MRV, and LMBV), and EHNV/ENARV/ATV/CMTV/FV3 (including EHNV, ENARV, ATV, CMTV, and FV3). In conclusion, the phylogenetic tree constructed from core genes demonstrates that MRV was classified within the *Ranavirus* genus, which comprises seven species and three sister lineages.

### Interspecies methylation differences in ranavirus

The role of methylation in differentiating ranavirus species remains uncertain, prompting our investigation. As shown in Fig 4C, the Singapore grouper iridovirus (SGIV) genome is considered unmethylated [1], primarily due to the absence of viral DNA methyltransferases (DNMTs). Our study found that the genomic methylation level of MRV was 16.04%, while Dawn BW et al. reported FV3’s level at 21.9% [12]. Although research on ranavirus genomic methylation levels is limited, the observed increasing distribution of ranavirus genomic methylation levels shows a correlation with the protein phylogenetic analysis. We further analyzed the DNMT protein sequence features in the genomes of ranaviruses. Among the seven prototypical ranavirus species, DNMT is present in all except SGIV [21]. The domain analysis within the DNMT of all viruses was showed that they contain a DNA-cytosine methylase domain (Fig 4D). Notably, the DNA-cytosine methylase domain of SCRAV (MRV) consists of 80 amino acids, whereas the domains of EHNV, ENARV, ATV, CMTV, and FV3 each have 114 amino acids. The similarity of the DNMT protein encoded by SCRAV (MRV) to those of the other five ranaviruses is below 70%, whereas the similarity among the DNMT proteins of these five viruses exceeds 90% (Fig 4E). Amino acid sequence homology comparison results showed that the DNMT of SCRAV (MRV) had slightly different amino acid sequences from the other five ranaviruses, while the DNMTs of EHNV, ENARV, ATV, CMTV, and FV3 were highly homologous (S2 Fig). In conclusion, DNMT variations among ranaviruses might support to the hypothesis of three distinct lineages: SGIV, SCRAV (MRV/LMBV), and EHNV/ENARV/ATV/CMTV/FV3. However, given the limited research on genomic methylation levels and DNMT variability within ranavirus genomes, we hypothesize that genomic methylation level distribution may still correlate with the phylogenetic tree; further validation is needed.

### Ranavirus whole-genome phylogenetic analysis

While a correlation appears to exist between the DNMT variations and the evolutionary dynamics of ranaviruses, this assessment is currently limited to the core gene level. To further investigate the interspecific evolutionary relationships among ranaviruses, we constructed and analyzed a phylogenetic tree based on complete genomic DNA sequences for the first time. 217 viral genomes from *Alphairidovirinae* were used to construct the nucleic acid phylogenetic tree (Fig 4F). Consistent with the protein phylogenetic tree, MRV belongs to the *Ranavirus* genus, and ranaviruses are classified into seven species: SGIV, SCRAV, EHNV, ENARV, ATV, CMTV, and FV3. The genetic subtypes of these species align with those observed in the protein phylogenetic tree. Interestingly, unlike the protein phylogenetic tree analysis, whole-genome phylogenetic analysis reveals four sister lineages of ranaviruses: SGIV, SCRAV (MRV/LMBV), EHNV/ENARV/ATV, and CMTV/FV3. Notably, the EHNV/ENARV/ATV/CMTV/FV3 lineage is divided into two sister lineages: EHNV/ENARV/ATV and CMTV/FV3.

### Collinearity analysis of the ranavirus genome

**Fig 5:**
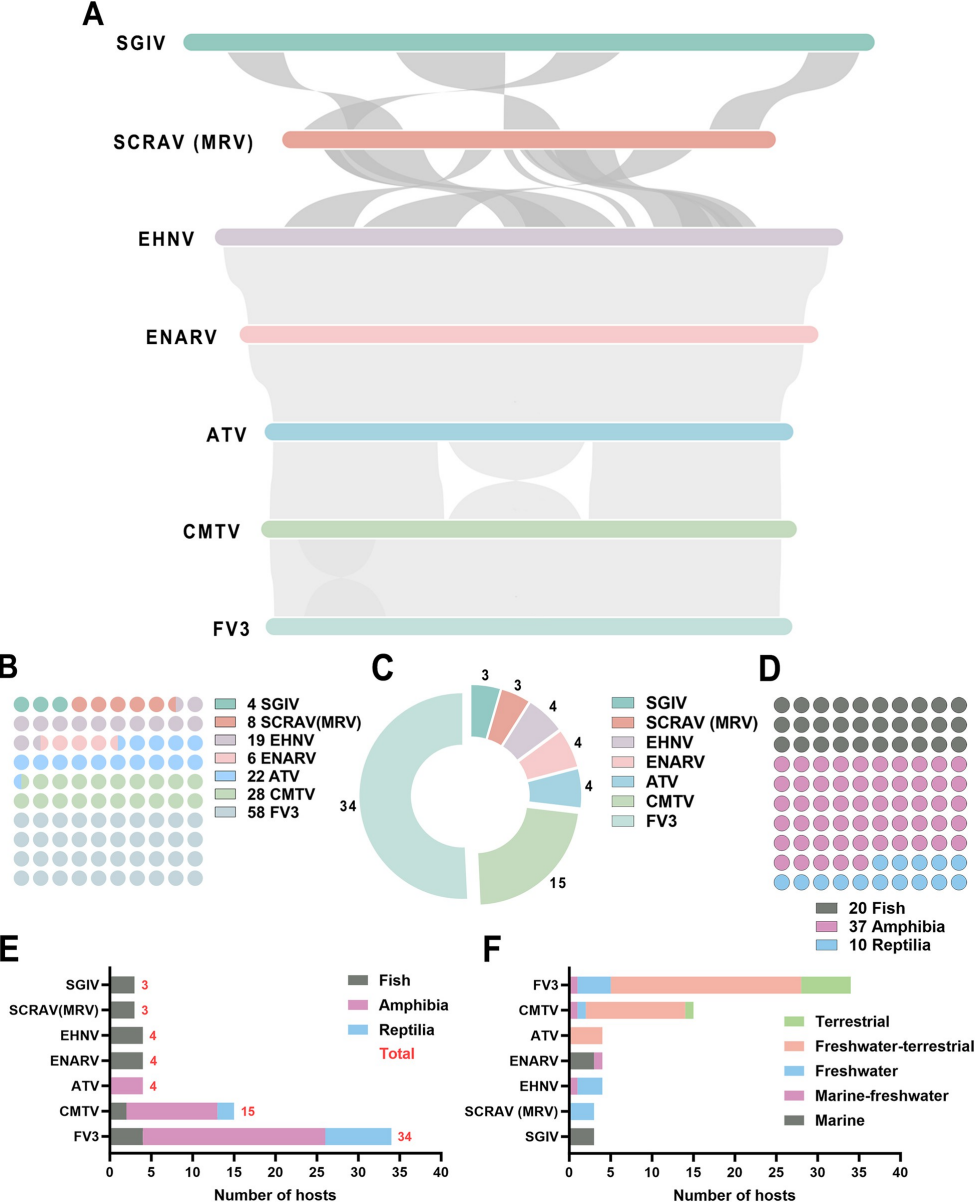
The relationship between ranaviruses and its natural host. (A) Genomic collinearity analysis of ranaviruses. The relevant accession numbers for the genomic data used in the analysis are: SGIV: NC_006549.1; MRV: MG941005.3; EHNV: NC_028461.1; ENARV: NC_075538.1; ATV: NC_005832.1; CMTV: NC_039034.1; FV3: NC_005946.1. (B) Quantitative assessment of isolates derived from seven prototypical species of ranaviruses. (C) The number of natural hosts for each species of ranaviruses. (D) Classification of the natural hosts of all ranaviruses. (E) Assessment of the biodiversity of the natural hosts of each ranavirus. (F) The habitat distribution of natural hosts for each ranavirus.

To further investigate interspecific evolutionary relationships within ranaviruses, we employed genomic collinearity analysis on seven representative species. Except for SCRAV, which was substituted with MRV (Accession number, MG941005.3), the genomes of all other viruses adhered to the reference genome suggested by ICTV. As shown in Fig 5A, the genomes of SGIV and MRV exhibited lower collinearity, with numerous instances of sequence inversions and rearrangements. Similarly, weak collinearity was observed between the SCRAV and EHNV genomes, accompanied by a substantial number of sequence inversions and rearrangements. However, a strong collinearity was observed among the EHNV, ENARV, and ATV genomes. Although the ATV and CMTV genomes generally showed strong collinearity, a noticeable phenomenon of sequence inversion was observed. lastly, a strong collinearity was identified between CMTV and FV3 genomes. These results suggested that ranaviruses may have undergone three gene sequence inversion and rearrangement events, and that there are four potential evolutionary lineages: SGIV, SCRAV (MRV/LMBV), EHNV/ENARV/ATV, and CMTV/FV3.

### Distribution of natural hosts of ranaviruses and its habitats

Based on whole-genome phylogenetic tree and genomic collinearity analyses, we speculated the existence of sister lineages for EHNV/ENARV/ATV and CMTV/FV3, although the extent of their divergence remains unclear. To investigate, we analyzed the distribution of natural hosts of ranaviruses and its and habitats. The analysis encompassed all 145 ranaviruses strains archived in the GenBank database, comprising 4 SGIV, 8 SCRAV (MRV), 19 EHNV, 6 ENARV, 22 ATV, 28 CMTV, and 58 FV3 strains (Fig 5B). After statistically de-duplicating natural hosts, we identified 67 species. All hosts included in the analysis were not artificially verified as susceptible hosts. As shown in Fig 5C, SGIV infects 3 species, SCRAV (MRV) infects 3, EHNV infects 4, ENARV infects 4, ATV infects 4, CMTV infects 15, and FV3 infects 34 species. All natural hosts are categorized as fish, amphibians, or reptiles, with amphibians exhibiting the highest diversity (37 species), followed by fish (20 species) and reptiles (10 species) (Fig 5D). The detailed species classification distribution for the seven prototypical ranavirus natural hosts was shown in Fig 5E. SGIV, SCRAV (MRV), EHNV, and ENARV primarily infect fish; ATV targets amphibians; CMTV and FV3 infect fish, amphibians, and reptiles, with the highest infection rates observed in amphibian. Reptiles are exclusively found in the CMTV/FV3 lineage.

**Fig 6:**
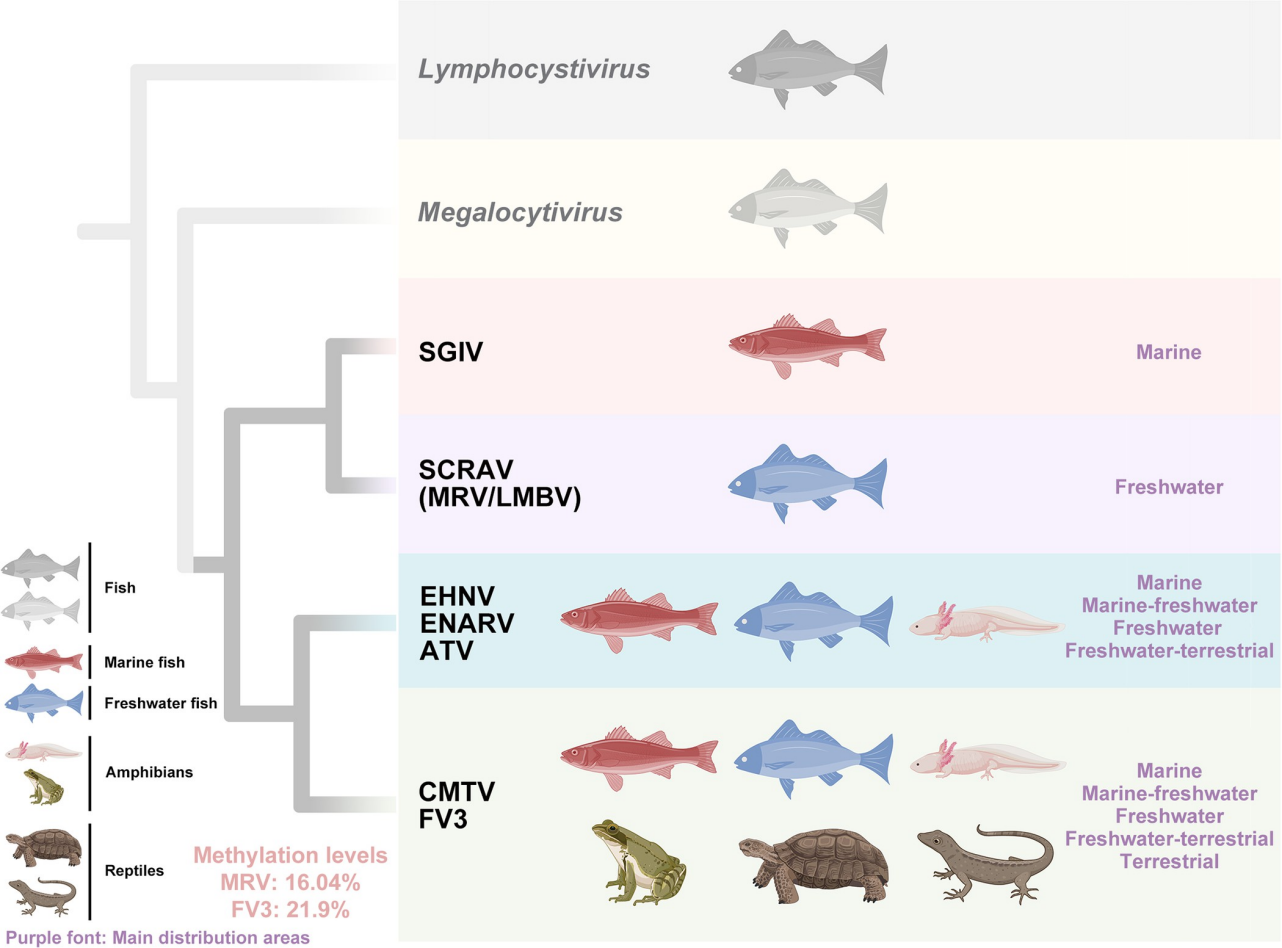
Hypothesis on the potential evolutionary relationships among ranaviruses. Four distinct evolutionary lineages "SGIV, SCRAV (MRV/LMBV), EHNV/ENARV/ATV, and CMTV/FV3" might be present in their evolution. The validity of this hypothesis might be supported by four potential evidences: whole-genome phylogenetic tree, genomic collinearity, natural host range and host habitats. We speculate that ranavirus genomic methylation levels might be another evidence for the hypothesis, but further proof is needed. Created in BioRender. PAN, W. (2024) BioRender.com/u29g202.

Furthermore, the natural hosts of ranavirus can be classified into five habitat categories: marine, marine-freshwater, freshwater, freshwater-terrestrial, and terrestrial (Fig 5F). The natural hosts of SGIV predominantly inhabit marine environments (hosts, *n* = 3); the natural hosts of SCRAV (MRV) are primarily in freshwater (hosts, *n* = 3); EHNV’s natural hosts are chiefly found in freshwater habitats (hosts, *n* = 3), with a minor presence in both marine and freshwater settings (hosts, *n* = 1); ENARV’s natural hosts are mainly associated with marine environments (hosts, *n* = 3), albeit with some occurrence in mixed marine and freshwater habitats (hosts, *n* = 1); ATV’s natural hosts exclusively thrive in freshwater and terrestrial ecosystems (hosts, *n* = 4). CMTV and FV3 exhibit comparable distribution patterns across habitat types, including marine-freshwater, freshwater, freshwater-terrestrial, and terrestrial zones. Nonetheless, notable differences exist species diversity: CMTV’s primary host populations are concentrated in freshwater-terrestrial zones (hosts, *n* = 12), while other categories remain relatively balanced. In contrast, FV3’s natural hosts predominantly occupy the freshwater-terrestrial zone (hosts, *n* = 23) followed by terrestrial-only habitats (hosts, *n* = 6), then pure freshwater systems (hosts, *n* = 4), concluding with marine-freshwater habitats (hosts, *n* = 1). It is worth noting that, excluding the shared freshwater environment, FV3’s natural hosts surpasses that of CMTV. These findings indicated that the habitats of hosts infected with the EHNV/ENARV/ATV lineage are inextricably linked to aquatic environments, while the habitats of hosts infected with the CMTV/FV3 lineage have expanded to include fully terrestrial habitats. These observations suggested that EHNV/ENARV/ATV and CMTV/FV3 might display independent evolutionary relationships. Therefore, based on the whole-genome phylogenetic tree, genomic collinearity, natural host distribution, and host habitat analysis results, we propose an evolutionary hypothesis: the existence of four distinct evolutionary lineages within ranavirus evolution, namely "SGIV, SCRAV (MRV/LMBV), EHNV/ENARV/ATV, and CMTV/FV3" (Fig 6).

## Discussion

DNA methylation is the process by which methyl groups are appended to cytosine or adenine. The methylation of 5mC plays a crucial role in gene activity in both eukaryotes and prokaryotes, influencing critical processes such as genomic imprinting, cell differentiation, transcriptional regulation, and chromatin remodeling [22, 23]. In this study, 16.04% of MRV genomes were subjected to methylation analysis via WGBS. In comparison to other ranaviruses, FV3 exhibits 21.9% methylation [12]. ISKNV and LCDV, two viruses belonging to the *Megalocytivirus* genus of the *Alphairidovirinae* family, showed 24% and 20% methylation, respectively [11, 24]. However, the genomic cytosine methylation levels of MRV were slightly lower, yet its genome remained hypermethylated. Notably, high cytosine methylation was not observed in the genomes of non-iridoviruses; for instance, only 0 to 1.7% of cytosine in the deno-associated virus type 2 genome were methylated [25], while the cytosine methylation levels of adenovirus type 2 (AAV2) and type 12 were 0.04 and 0.06%, respectively [26]. In vertebrates, the methylation level was reported to be 6.44% in the genome of the *S*. *chuatsi* [27] and 6.2% in the genome of the fathead minnow cells [12]. In mammals, the level of methylation stands at approximately 5% in the human genome [28], 5.1% in the pig genome [29], and 2.22% in baby hamster kidney fibroblast cells [26]. Furthermore, our study has further elucidated the genomic methylation profile of MRV. Specifically, it has been found that the DNA across the MRV virion genome is methylated at 70.48% of all CpG sites. Meanwhile, the cytosine residues at the CA, CT, and CC sites exhibit minimal methylation or possess exceedingly low levels of methylation. The prevalence of hypermethylation is primarily observed at CpG sites and remains unaffected by the position of the site or by the nucleotide at the 3’ end of the site. Notably, CpG methylation appears to be correlated with the nucleotide at its 5’-terminal 1 bp position. When a cytosine follows the CpG at the 5’-terminal 1 bp position, the CpG methylation level surges to 94.21%, exceeding the average CpG methylation level by over 20%. Regarding the other three contexts, the methylation levels of ACG, TCG, and GCG were recorded as 70.62%, 52.08% and 59.54%, respectively. Whether this is a coincidence or attributable to other factors is uncertain, as MRV’s DNMT diverges significantly from other five ranaviruses. This discrepancy’s origin, possibly from DNMT preferences, requires further investigation. In summary, the genomic DNA of MRV exhibits hypermethylation, primarily at CpG sites, and the correlation between CpG methylation and 5’-terminal nucleotide type remains ambiguous, warranting further validation.

Methylation plays a pivotal role in the replication processes of viruses. Upon infection, the methylation levels of the genomes of human papillomavirus [30], hepatitis B virus [31], and AAV2 [25] are elevated, enabling evasion of the host’s immune system. Similarly, hypermethylation of the genomic DNA of ISKNV, a megalocytivirus, facilitates evasion of the host’s innate immune response [11]. It is generally acknowledged that, apart from SGIV, the viral genomes of the *Alphairidovirinae* iridoviruses are extensively methylated and encode a DNA methyltransferase that catalyzes methylation of cytosine reduces with CpG dinucleotides [1]. Our analysis of the MRV genome confirmed that it also encodes a DNA methyltransferase. The packaging of DNA into virions is significantly influenced by methylation. Evidence indicates that FV3 encodes a DNA methyltransferase that methylates FV3 DNA within the cytoplasm [32]. The FV3 DNA methylase demonstrates the capability to methylate a range of natural and synthetic DNA *in vitro* [33] and possesses endonuclease activity. When the FV3 DNA methylase is deleted, the virus remains viable yet lacks viral endonuclease activity [32]. In other ranaviruses, including MRV, DNA methyltransferases have been inferred solely from their genomes, with their precise functions remaining unexplored. In this study, we have demonstrated that the genomic methylation of MRV can be suppressed through treatment with 5-Azacytidine, leading to the inhibition of MRV replication. MRV-Aza, a virus with hypomethylated genomic DNA due to 5-Azacytidine treatment, has reduced replication capacity compared to wild-type MRV. Genomic hypomethylation decreased the mortality rate of *S*. *chuatsi* infected by MRV, a phenomenon also observed in ISKNV [11]. These results suggested genomic methylation may be important for MRV replication; however, 5-Azacytidine integrates into DNA while inhibiting DNA methylation [19], and its impact on MRV replication is unclear. In summary, 5-Azacytidine treatment effectively reduces MRV DNA methylation levels and inhibits its replication, and the replication of the MRV-Aza virus with low genomic methylation is attenuated. We speculated that genomic demethylation could affect MRV replication; however, it is uncertain if these findings are related to 5-Azacytidine incorporation into DNA, warranting further investigation.

Ranavirus is an emerging infectious disease associated with mass mortality events across various amphibian species [34]. Given the wide range of ranaviruses, encompassing ecologically and economically important species, understanding their evolution, including unique genomic rearrangements linked to host specificity and viral evolution, is crucial for predicting and potentially preventing further ranaviruses epidemics in animals [35]. Previous studies have primarily conducted phylogenetic analyses of ranaviruses using single genes (e.g., MCP) [36,37] or multiple genes [15]. Although analyzing the MCP gene is convenient, its highly conservation may obscure differences between virus isolates. This study conducted a whole-genome DNA sequence phylogenetic analysis of ranaviruses to accurately classify and type the viruses, encompassing seven species and eleven genotypes. Compared to studies on the spread of ranaviruses, fewer have focused on their evolution [5,15]. This study proposes, for the first time, a potential interspecific evolutionary relationship among "SGIV, SCRAV(MRV/LMBV), EHNV/ENARV/ATV, and CMTV/FV3" in ranaviruses. The validity of this hypothesis might be supported by four lines of potential evidence: whole-genome phylogenetic tree, genomic collinearity, natural host range and host habitats. The whole-genome phylogenetic tree analysis four evolutionary branches within ranavirus populations; Genomic collinearity analysis shows a sequence inversion phenomenon between the EHNV/ENARV/ATV lineage and the CMTV/FV3 lineage; The phylogenetic lineage differentiation is also reflected in the host species, with SGIV and SCRAV (MRV/LMBV) primarily infecting fish, EHNV/ENARV/ATV infecting fish and amphibians, and CMTV/FV3 capable of infecting fish, amphibians, and reptiles. Similarly, categorizing hosts by their primary habitats reveals distinct characteristics among the four lineages. The SGIV lineage is predominantly associated with marine environments, while the SCRAV (MRV/LMBV) lineage is primarily found in freshwater ecosystems. The EHNV/ENARV/ATV lineage occupies marine, freshwater, and terrestrial habitats, with all hosts fundamentally linked to aquatic environments. However, the CMTV/FV3 lineage also inhabits marine, freshwater, and terrestrial settings but includes completely terrestrial hosts. In addition, ranavirus genomic methylation levels may provide additional evidence for this hypothesis, but further proof is needed. In the whole-genome phylogenetic tree, the genus *Megalocytivirus* was identified as the closest sister group to genus *Ranavirus*. All natural hosts of megalocytiviruses are fish [1], and similarly, all four sister lineages of ranaviruses might also have natural fish hosts (Fig 6). This leads to the speculation that the ancestral lineage of ranaviruses may have originated from a fish virus. These will advance our understanding of ranaviruses and contribute to the prevention of ranavirus transmission and infection. Notably, according to our proposed evolutionary relationship of ranaviruses, their hosts might also shift from seawater to freshwater to land. Nowadays, human activities are intensifying species evolution [38] and extinction [39], and it cannot be ruled out that in the future, ranaviruses will evolve to infect a broader level of animal hosts, thus necessitating increased attention and research on ranaviruses.

In summary, our study comprehensively characterized the whole-genome methylation of MRV and preliminarily elucidated that 5-Azacytidine can inhibit MRV genome methylation. This work is poised to advance in the field of viral epigenetics and enhance our comprehension of how ranaviruses harness methylation mechanisms. Furthermore, a hypothesis on the interspecific evolutionary relationships of ranaviruses has been proposed, providing novel insights into the evolutionary trajectories of ranaviruses.

## Materials and methods

### Ethics statement

All experimental procedures were approved by the Institutional Animal Care and Use Committee, Sun Yat-sen University (protocol code: SYSU-IACUC-2024-B0787, date of approval: April 2024). We have complied with the guide for the Care and Use of Laboratory Animals of Sun Yat-sen University.

### Cell and virus

Mandarin fish fry-1 (MFF-1) cells were cultured in Dulbecco’s modified Eagle medium (DMEM; GIBCO, USA) supplemented with 10% fetal bovine serum (FBS; Hyclone, USA) and maintained in a humidified atmosphere at 27°C with 5% CO_2_ [40]. In 2016, the MRV strain, named NH-1609 (Accession number: MG941005), was isolated from diseased mandarin fish in a farm in Nanhai District, Foshan, Guangdong Province, and was subsequently stored in our lab [41]. The MRV strain was then inoculated into MFF-1 cells at a multiplicity of infection (MOI) of 0.001. Upon the manifestation of CPE, the cells were harvested and stored at −80°C for future use.

### Extraction of the genome DNA from purified MRV virions

Upon observing CPE in the MFF-1 cells, the culture medium was subjected to three freeze-thaw cycles at -80°C. Virions were purified using a modified double sucrose gradient ultracentrifugation method, as previously described [42]. Subsequently, genomic DNA was extracted from the virions using the FastPure Cell/Tissue DNA Isolation Mini Kit (Vazyme, China), and its integrity and concentration were evaluated using agarose gel electrophoresis and a NanoDrop 2000 spectrophotometer (Thermo Fisher Scientific, USA).

### Bisulfite conversion and sequencing

The qualified DNA (*n*  =  4 biological replicates) was subjected to crucial steps for sequencing, including fragmentation, terminal repair, A-tail addition, splice addition, fragment screening, bisulfite treatment, and PCR amplification. These steps culminated in the generation of DNA libraries suitable for sequencing on the NovaSeq 6000 (PE150) platform (Illumina, USA). Library construction and sequencing were expertly performed by Annoroad Gene Technology (Beijing) Co., Ltd. in Beijing, China. The raw data underwent meticulous trimming and filtering using Trimmomatic v0.39 (ILLUMINACLIP:TruSeq3-PE-2:2:30:10:8:true SLIDINGWINDOW:5:20 LEADING:5 TRAILING:5 MINLEN:36) [43], with quality control further boosted through FastQC v0.12.1 (https://www.bioinformatics.babraham.ac.uk/projects/fastqc/). Subsequently, the clean data were mapped to the MRV genome (Accession number: MG941005.3), deduplicated, and methylation patterns were extracted using Bismark v0.24.2 [44] with the "--ignore_r2 2" option. Among them, λDNA was mixed into MRV genome samples as a completely unmethylated negative control to confirm the conversion rate of bisulfite treatment [45].

### Analysis of CpG-dense regions

The analysis of CpG-dense regions in MRV was conducted utilizing the EMBOSS Cpgplot and the EMBOSS Newcpgreport tools available at EMBL−EBI [46].

### Absolute quantitative PCR (qPCR)

Cells were cultured in 24-well plates for viral replication assays. Following treatments and MRV infection, the cellular DNA was extracted using the DNA Isolation Mini Kit (Vazyme, China). To quantify MRV genome copies, a standard curve was generated using a series of diluted pCMV-n-myc-mrv*MCP* plasmids. Subsequently, genome copies were measured using 2× Real PCR Easy Mix-TaqMan (Foregene, China) based on the standard curve. The reaction commenced with denaturation at 95°C for 1 min, followed by 45 cycles of denaturation at 95°C for 15 s, annealing at 60°C for 30 s, and extension at 72°C for 1 s. The gene-specific primers used are listed in S4 Data.

### RNA extraction and quantitative PCR for reverse transcription (RT-qPCR)

Cells were infected with MRV at an MOI of 0.01. After 2 h, the medium was replaced with fresh DMEM containing 10% FBS, and the cells were treated with either DMSO (MP Biomedicals, USA) or 5-Azacytidine (MedChemExpress, USA). Cells were collected at 24, 48, and 72 h post-infection. RNA was extracted from the cell samples using the Eastep Super Total RNA Extraction Kit (Promega, China). cDNA was synthesized from 200 to 500 ng of total RNA using *Evo M-MLV* RT Premix for qPCR kit (Accurate Biology, China). RT-qPCR was performed using the SYBR Green Premix *Pro Taq* HS qPCR Kit II (Accurate Biology, China) on a Roche LightCycler 480 System, employing gene-specific primers as described in S4 Data. The cycling conditions comprised an initial denaturation at 95°C for 30 s, followed by 40 cycles of 95°C for 5 s, 60°C for 20 s, and 70°C for 20 s. A final cycle from 95°C to 5°C was performed to generate a melting curve. To normalize the mRNA levels, the expression of *S*. *chuatsi β-actin* served as the reference standards. Ultimately, the expression levels of the target mRNAs were calculated using the 2^−ΔΔCT^ quantification method.

### Methylation-specific PCR (MSP) and bisulfite sequencing PCR (BSP)

DNA was extracted from the samples using the DNA Isolation Mini Kit (Vazyme, China), followed by bisulfite modification of the DNA using a DNA Bisulfite Conversion Kit (Tiangen, China). Subsequently, the modified DNA was amplified using EpiTaq HS (TaKaRa, China) according to the manufacturer’s protocol. The amplification program consisted of 35 cycles, with each cycle including denaturation at 98°C for 10 s, annealing at 55°C for 30 s, and extension at 72°C for 30 s. For MSP, the reaction products were electrophoresed, and the resulting image was captured using the Tanon MINI Space 2000. Conversely, for BSP, the modified DNA was amplified following the same protocol as MSP, with the employment of different primers. The amplified DNA fragments were then cloned into the pMD19-T vector (TaKaRa, China), and the recombinant vectors were subsequently sequenced. The sequencing results were analyzed and compared using DNAMAN 10.0. The specific primers employed in this process are detailed in S4 Data.

### Preparation of polyclonal antibody

The nucleic acid fragment corresponding to amino acids from 17 to 144 of the mrv*ORF097L* open reading frame (ORF) peptide sequence was amplified from MRV DNA using primers mrv*ORF097*-F and mrv*ORF097-*R (S4 Data). The target PCR product was ligated into the pcDNA3.1/Myc-His(−) vector using the ClonExpress II One Step Cloning Kit (Vazyme, China) to obtain the recombinant expression vector. This recombinant plasmid, pET-mrv*ORF097L*, was transformed into competent *Escherichia coli* BL21 (DE3), and the recombinant protein was purified using the HisBind Purification Kit (Novagen, Germany) following the manufacture’s protocol. Two New Zealand rabbits each received four subcutaneous injections of a mixture containing 2 mg of purified mrv*ORF097L* recombinant protein and either Freund’s complete adjuvant (FCA) or Freund’s incomplete adjuvant (FIA). FCA was utilized solely for the first injection, while FIA was used for the subcutaneously injections. The second injection was performed 28 days after the first injection, and the remaining injections were administered at two-week intervals. Antiserum titers were measured by ELISA three days after the fourth injection, and the rabbit with the highest titer was sacrificed two weeks later for serum collection. The specificity of anti-mrvORF097L serum was verified by western blotting (WB) using samples of cells that were either infected or not infected with MRV, following a previously described WB method [47]. The anti-mrvORF097L serum was stored at −80°C until needed. This animal work was approved by the Institutional Animal Care and Use Committee of Sun Yat-sen University, with the approval number SYSU-IACUC-2024-B0708.

### WB assay

Cells infected with MRV were collected 72 h post-infection and lysed in 200 μL of WB (IP) Lysis Buffer (Abbkin, China). The protein concentration was then determined using a BCA protein quantification kit (Beyotime, China). Equal amounts of protein samples, after adding Omni-Easy Protein Sample Loading Buffer (Epizyme, China), were separated on 4–20% FastPAGE (TSINGKE, China) under 180 V for 30 min. Subsequently, the proteins were transferred electrophoretically to a 0.22 μm PVDF membrane (Millipore, USA). The anti-mrvORF097L polyclonal antibody, diluted to an appropriate concentration (1:1000–2000), was used as the primary antibody to detect the viral protein. Glyceraldehyde-3-phosphate dehydrogenase (GAPDH) from *S*. *chuatsi* served as a reference protein and was detected using a rabbit anti-GAPDH antibody (Abways, China). Goat anti-rabbit IgG (H+L) (Promega, USA) was used as the secondary antibody. Finally, images were acquired using an Amersham ImageQuant 800 imaging system with a Chemistar High-sig ECL Western Blotting Substrate kit (Tanon, China).

### Animal challenge

The one-month-old mandarin fish (*S*. *chuatsi*), with an average weight of 30 ± 5 g, were collected from a commercial fish farm in Foshan, Guangdong Province, China. Prior to the experiment, six juvenile fish were randomly selected and confirmed via conventional PCR to be free of viral infections. Subsequently, all fish were acclimated for 7 days before being randomly divided into groups, each containing twenty-five fish. Two groups of fish were intraperitoneally injected with 0.1 mL of different titers (10^7.625^ TCID_50_/fish) of wild-type MRV or hypomethylated MRV, while another group received 0.1 mL of sterile DMEM as a negative control. The fish were fed and monitored daily, and the survival rates were calculated until there were no fish deaths for five consecutive days.

### Evolutionary tree construction, protein domain analysis, and collinearity analysis

Nucleic acid sequences of 217 iridovirus strains of *Alphairidovirinae* were retrieved from the GenBank database [48] (viruses are listed in S3 Data). To eliminate the influence of terminally redundant sequences [1] and potential reverse complementarity on subsequent analysis when the genomic sequences were uploaded to the database, the position of the terminally redundant sequences were adjusted and/or the genomic sequences were reversed complemented. All genome sequences were corrected to ensure that the putative major coat protein (MCP) gene was the first gene in the genome sequence (5’−3’), and all subsequent analyses were performed on the corrected genome sequence. The Pairwisealigner of Bio-align package in Biopython v1.80 [49] was used to globally align the viral genomic sequences and obtain pairwise sequence identity.

For the nucleic acid phylogenetic tree, IQ-TREE v2.3.4 [50] was employed to construct the phylogenetic tree with model selection [51] and ultrafast bootstrap (UFBoot) [52] using the parameters "-m MFP+LM -B 1000 -bnni", following multiple alignment of the corrected viral genomic sequences using the FFT-NS-2 algorithm in MAFFT v7.526 [53]. For the core/specific proteins phylogenetic trees, twenty-six iridovirus core genes and twenty-seven ranavirus-specific genes were reported by Eaton HE et al. [54] and Jancovich JK et al. [15]. For the protein phylogenetic trees, the iridovirus core genes of 214 iridovirus strains of *Alphairidovirinae* and the ranavirus-specific genes of 145 ranavirus strains were obtained using ORFfinder [55] and BLAST+ v2.15.0 [56]. IQ-TREE was used to construct the phylogenetic trees with partition model [57], ModelFinder [58] and UFBoot with the parameters "-m MFP+MERGE -B 1000 -bnni" after performing multiple alignment of the protein sequences using the L-INS-i algorithm in MAFFT. For the DNMT phylogenetic tree, IQ-TREE was used to construct the phylogenetic trees with model selection and UFBoot with the parameter "-m MFP -B 1000 -bnni" after performed multiple alignment of the protein sequences using L-INS-i algorithm in MAFFT. Phylogenetic trees were visualized using iTOL [59]. Based on the classification of ISKNV genotypes by Kurita J et al. [60] and Fusianto C et al. [61], the genotypes of ISKNV, FV3, epizootic haematopoietic necrosis virus (EHNV), and common midwife toad virus (CMTV) were classified according to the phylogenetic trees. For protein domain analysis, CD-search [62] was used for analysis with default parameters. For gene synteny and collinearity analysis, BLAST+ and MCScanX [63] were used with the parameter "-s 3". Visualization of gene synteny and collinearity were performed using famCircle v0.2.6 (https://github.com/lkiko/famCircle).

### Statistical analysis

To minimize errors, all experiments were conducted with a minimum of three replicates. Statistical analysis of the data involved one-way analysis of variance (ANOVA), followed by a two-tailed Student’s *t*-test for further comparisons. The outcomes were articulated as the mean ± standard error of the mean (SEM), and *p*-values less than 0.05 and 0.01 were considered statistically significant and extremely significant differences, respectively. Both GraphPad Prism 10.1.2 (GraphPad Software, USA) and SPSS 29.0.1 (IBM, USA) were employed throughout the entire data analysis process.

## Supporting information

S1 Fig MSP analysis for three regions within the MRV and MRV-Aza genomes. (TIF)

S2 Fig Homology alignment analysis of DNA methyltransferases in prototypical species of ranaviruses. The analysis was performed utilizing DNAMAN 10.0.
(TIF)

S1 Data ORFs in the MRV genome. (XLSX)

S2 Data CpG dense regions in the MRV genome. (XLSX)

S3 Data Virus grouping. (XLSX)

S4 Data Primers in this study. (XLSX)

S5 Data Methylation landscape of the MRV genome. (XLSX)

S6 Data Numerical source data. (XLSX)

S7 Data Viruses and natural hosts. (XLSX)
