# Supplementary figures and images for: Viral genomic methylation and the interspecies evolutionary relationships of ranavirus
Source: PLoS Pathog. 2024 Nov 25;20(11):e1012736. doi: 10.1371/journal.ppat.1012736 (PMC11627377; doi:10.1371/journal.ppat.1012736)

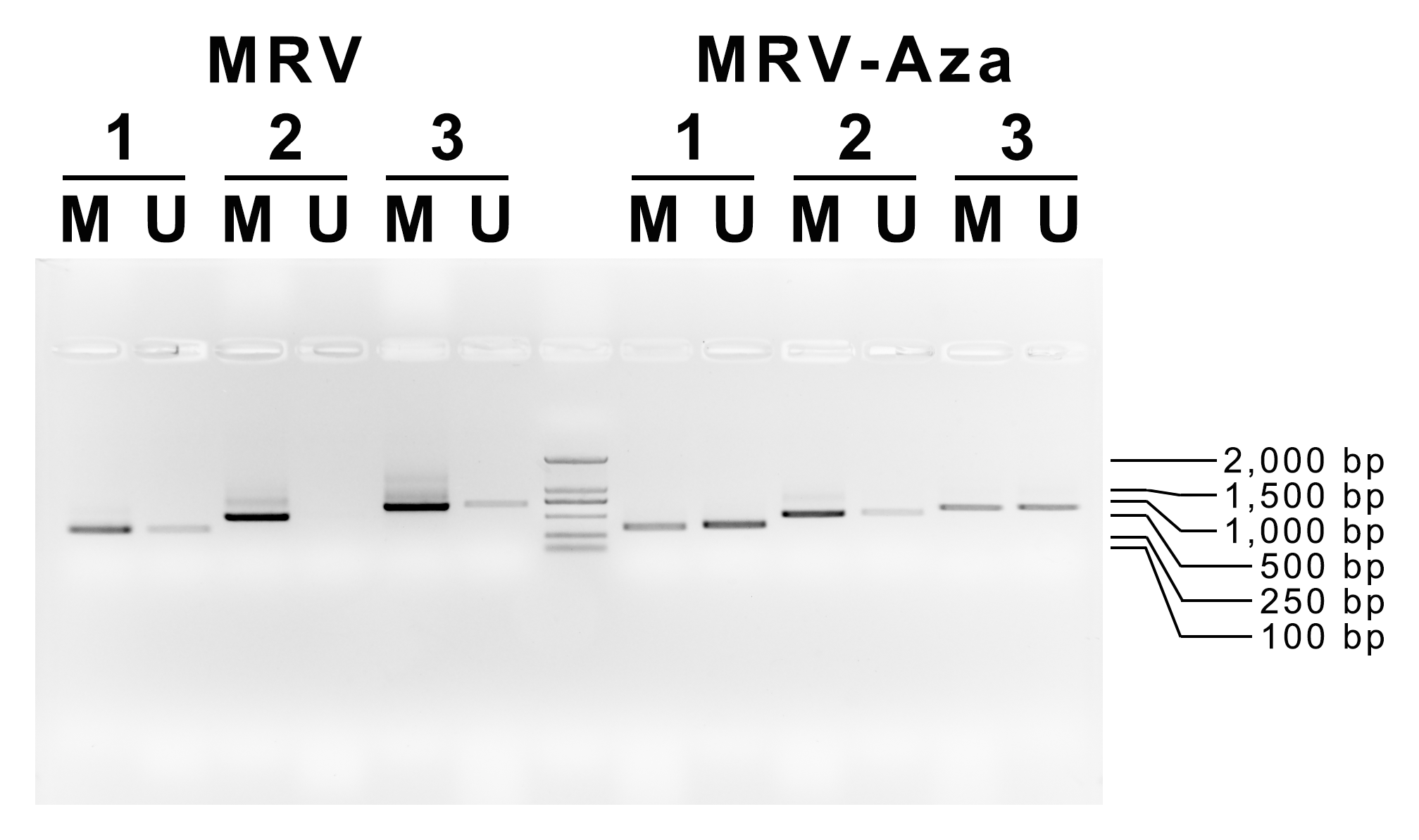

Supplement: S1 Fig — (TIF) [file ppat.1012736.s001.tif]

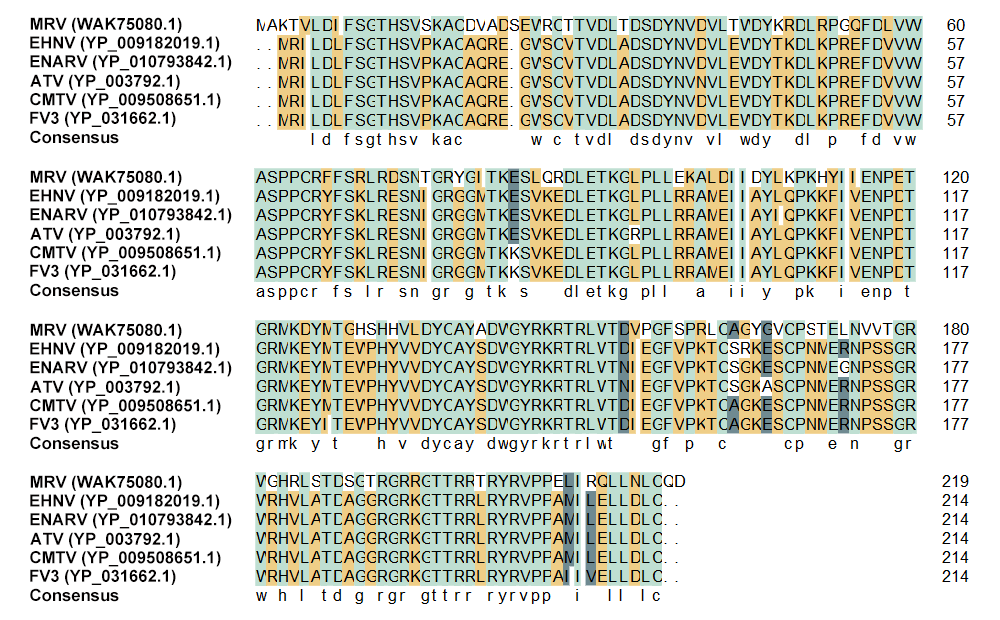

Supplement: S2 Fig — The analysis was performed utilizing DNAMAN 10.0. (TIF) [file ppat.1012736.s002.tif]
